# Long-term exposure to diesel exhaust particles induces concordant changes in DNA methylation and transcriptome in human adenocarcinoma alveolar basal epithelial cells

**DOI:** 10.1186/s13072-024-00549-3

**Published:** 2024-08-05

**Authors:** Alexandra Lukyanchuk, Naomi Muraki, Tomoko Kawai, Takehiro Sato, Kenichiro Hata, Tsuyoshi Ito, Atsushi Tajima

**Affiliations:** 1 Department of Bioinformatics and Genomics, Graduate School of Medical Sciences, Kanazawa University, 13-1 Takara-machi, Kanazawa, 920-8640 Japan; 2 Krasnoyarsk State Medical University Named After Prof. V.F. Voino-Yasenetsky, Krasnoyarsk, Russia; 3 Health Effects Research Group, Environment Research Division, Japan Automobile Research Institute, Tsukuba, Japan; 4 Department of Maternal-Fetal Biology, National Research Institute for Child Health and Development, Tokyo, Japan; 5 Department of Human Biology and Anatomy, Graduate School of Medicine, University of the Ryukyus, Nishihara, Japan; 6 Department of Human Molecular Genetics, Gunma University Graduate School of Medicine, Maebashi, Japan

**Keywords:** Diesel exhaust particles, Alveolar epithelium, Long-term exposure, Methylation profiling, Gene expression profiling

## Abstract

**Background:**

Diesel exhaust particles (DEP), which contain hazardous compounds, are emitted during the combustion of diesel. As approximately one-third of the vehicles worldwide use diesel, there are growing concerns about the risks posed by DEP to human health. Long-term exposure to DEP is associated with airway hyperresponsiveness, pulmonary fibrosis, and inflammation; however, the molecular mechanisms behind the effects of DEP on the respiratory tract are poorly understood. Such mechanisms can be addressed by examining transcriptional and DNA methylation changes. Although several studies have focused on the effects of short-term DEP exposure on gene expression, research on the transcriptional effects and genome-wide DNA methylation changes caused by long-term DEP exposure is lacking. Hence, in this study, we investigated transcriptional and DNA methylation changes in human adenocarcinoma alveolar basal epithelial A549 cells caused by prolonged exposure to DEP and determined whether these changes are concordant.

**Results:**

DNA methylation analysis using the Illumina Infinium MethylationEPIC BeadChips showed that the methylation levels of DEP-affected CpG sites in A549 cells changed in a dose-dependent manner; the extent of change increased with increasing dose reaching the statistical significance only in samples exposed to 30 µg/ml DEP. Four-week exposure to 30 µg/ml of DEP significantly induced DNA hypomethylation at 24,464 CpG sites, which were significantly enriched for DNase hypersensitive sites, genomic regions marked by H3K4me1 and H3K27ac, and several transcription factor binding sites. In contrast, 9,436 CpG sites with increased DNA methylation levels were significantly overrepresented in genomic regions marked by H3K27me3 as well as H3K4me1 and H3K27ac. In parallel, gene expression profiling by RNA sequencing demonstrated that long-term exposure to DEP altered the expression levels of 2,410 genes, enriching 16 gene sets including *Xenobiotic metabolism*,* Inflammatory response*, and *Senescence*. *In silico* analysis revealed that the expression levels of 854 genes correlated with the methylation levels of the DEP-affected *cis-*CpG sites.

**Conclusions:**

To our knowledge, this is the first report of genome-wide transcriptional and DNA methylation changes and their associations in A549 cells following long-term exposure to DEP.

**Supplementary Information:**

The online version contains supplementary material available at 10.1186/s13072-024-00549-3.

## Background

Diesel exhaust particles (DEP) are an air pollutant consisting of a mixture of a carbonaceous core and various compounds such as aldehydes and polycyclic aromatic hydrocarbons. Due to the presence of toxic elements (e.g. benzo(a)pyrene, fluoranthene, benzo(a)anthracene) in DEP, research on the effects of DEP on organisms has been conducted for more than 20 years. Studies on human, animal, and cell samples exposed to DEP have reported oxidative stress [1–3], pro-inflammatory responses [4, 5], and reduced lung function with long-term exposure [6, 7]. In particular, mice repeatedly exposed to DEP for 8 weeks exhibited fibrotic foci and significantly higher levels of α-smooth muscle actin and fibronectin in their lungs, indicating the rapid progression of pulmonary fibrosis [7]. Such a finding suggests that long-term DEP exposure disrupts normal airway functions; however, the mechanisms behind the effects of DEP on the respiratory tract are still under investigation.

The adverse effects of chemicals on the respiratory tract are usually assessed by determining transcriptional alterations in cells derived from different regions of the respiratory tract, including the nasopharyngeal, tracheobronchial, and alveolar regions. For instance, the long-term effects of DEP on genome-wide transcriptional changes in mouse [8] and human [9] nasal epithelium cells have been investigated; moreover, the transcriptome of human bronchial epithelial cells after 15 weeks of DEP exposure has been described by Rynning et al. [10]. However, genome-wide transcriptional changes in alveolar epithelial cells due to long-term DEP exposure have not yet been investigated.

The alveolar epithelium consists of alveolar epithelial type I (ATI) and type II (ATII) cells separated by inter-alveolar septa. ATI cells cover 90% of the alveolar surface and perform barrier and gas exchange functions, while ATII cells reduce surface tension, regulate innate immunity in the lungs, and repair injured alveoli by differentiating into ATI cells [11]. Repeated alveolar epithelial injury as well as the abnormal regenerative response and senescence of ATII cells are the primary causes of edema, acute respiratory distress syndrome, and pulmonary fibrosis [12–14]. Thus, it is important to determine the specific effect of long-term DEP exposure on ATII cells. However, the main obstacle in performing long-term experiments on this type of cells is their tendency to spontaneously transdifferentiate into ATI-like cells [15, 16]. To overcome this limitation, we first focused on the effects of DEP on A549 cells. These cells are frequently employed as a model for ATII cells, including for epigenetic studies [17].

DNA methylation, which is associated with gene expression, is an important factor influencing pathological changes in alveolar epithelium, leading to the development of lung cancer [18], pulmonary fibrosis [19], and asthma [20]. Several studies have shown that short-term DEP exposure is capable of altering the epigenetic pattern of human bronchial epithelial cells [21–23]. Zhang et al. [22] comprehensively investigated the relationship between DNA methylation changes and gene expression in human bronchial epithelial cells. They reported that 24-h exposure to DEP resulted in increased expression of *TET1*, *DNMT1* and *DNMT3A* genes, which are possibly associated with DNA methylation alterations. Additionally, changes in DNA methylation levels induced by DEP exposure were located at transcription factor binding sites (TFBSs) and co-localized with histone markers for promoters, enhancers, and actively transcribed gene bodies. However, the cellular reactions induced by long-term DEP exposure may differ from those caused by short-term exposure. At present, there are no published data on the DNA methylation changes in alveolar epithelial cells under long-term DEP exposure.

Hence, we conducted this study to determine the effects of prolonged DEP exposure on the DNA methylome and transcriptome of human adenocarcinoma alveolar basal epithelial A549 cells. We first determined the DEP dose sufficient to induce significant DNA methylation changes after four weeks of exposure. The effect of DEP at the identified dose was then assessed by genome-wide screening of DNA methylation and gene expression levels. To understand the functional significance of DEP-induced DNA methylomic and transcriptomic changes, we performed *in silico* overrepresentation analysis with several known gene sets and analyzed the association between the DEP-induced changes and transcriptional regulatory domains such as epigenetic marks and TFBSs in A549 cells. Finally, we assessed whether DNA methylation and transcriptional changes could be correlated. This study provides baseline data on the long-term impact of DEP exposure on the DNA methylome and transcriptome of A549 cells and identifies potential avenues for further investigation of DEP effects on alveolar epithelial cells.

## Methods

### General study design

In the dose-response experiment, A549 cells were treated with three doses of DEP for four weeks, using 2–3 biological replicates per group. The DNA methylation levels of 850 K CpG sites were assessed for each treated group, and a sufficient dose of DEP was identified based on the results of differentially methylated analyses compared to the control.

In the main experiment, A549 cells were treated with the previously identified DEP dose for four weeks in four biological replicates, independent of the dose-response experiment. Differential methylation and differential expression analyses were used to estimate methylation levels of 850 K CpG sites and genome-wide transcriptional changes, respectively. The biological effects of DEP exposure on the methylome were evaluated by colocalizing differentially methylated CpG sites with other epigenetic marks and transcription factor binding sites. The impact of transcriptional changes was evaluated by assigning differentially expressed genes to the 51 gene sets representing specific, well-defined biological states or processes, as well as the ENCODE transcription factor target gene sets. To assess the coordination of DNA methylation and transcriptional changes, correlations were calculated between the DNA methylation level at individual differentially methylated *cis-*CpG sites and the expression level of the corresponding differentially expressed gene.

### DEP collection and preparation

The same batch of DEP as used in the previous study [24] was used for all subsequent experiments. To briefly describe the DEP preparation, a 3-L common rail direct injection diesel engine with oxidation catalyst and exhaust gas recirculation system was used to generate DEP. The engine was operated at 2,040 rpm and 35 Newton-meters on a 220 kW ECDY dynamometer (Meiden-Sya, Tokyo, Japan) using a fuel (LS light oil; ENEOS, Tokyo, Japan) containing 27 ppm sulfur and lubricating oil. DEP were collected from the accumulation in the dilution tunnel of the diesel exhaust generating system. The particle size distribution of the DEP based on particle number was analyzed with a scanning mobility particle sizer (model 3938; TSI, Inc., MN, USA), and the particle size was normally distributed with a peak at 100 nm in diameter and a maximum value of less than 800 nm. To measure the concentrations of polycyclic aromatic hydrocarbons (PAHs) in the DEP used, the DEP were subjected to Soxhlet extraction with dichloromethane. The soluble fraction of the DEP was about 10%. The contents of PAHs in the soluble fraction of the DEP were analyzed by fluorescence high-performance liquid chromatography (Shimadzu, Kyoto, Japan), and the concentration of each measurable PAH such as fluoranthene in the DEP was calculated (Additional file 1: Table S1). DEP were stored at − 80 °C until the experiments were performed. The stored DEP were thawed before use and suspended in DMEM/F12 medium containing 10% fetal bovine serum (FBS) and 10 µg/ml gentamicin) for cell exposure.

### Cell culture and treatment

A549 cells (ATCC, Manassas, VA, USA) were cultured in flasks containing DMEM/F12 medium supplemented with 10% FBS and 10 µg/ml gentamicin under humidified 5% CO_2_/95% air at 37 °C. At cell passages, cells adhering to the bottom of the flasks were detached with 0.25% trypsin/1 mM EDTA solution (Invitrogen, Carlsbad, CA, USA). A549 cells were seeded in T-25 cell culture flasks at a cell density of 0.25 × 10^5^ cells/ml and then exposed to DEP-suspended culture medium containing DEP at concentrations of 0, 3, 10, or 30 µg/ml. After one week, cells (at a density of 3–5 × 10^6^ cells/ml) were collected with the trypsin/EDTA solution and reseeded into a new T-25 cell flask at a density of 0.25 × 10^5^ cells/ml. The reseeding procedure was repeated a total of three times over a four-week DEP exposure period. The DEP-suspended culture medium was replaced at the time of reseeding and four days after seeding/reseeding. After four weeks of DEP exposure, cells were harvested for use in subsequent experiments.

### DNA isolation and DNA methylation arrays

DNA isolation was performed from each of the A549 cells exposed to different concentrations of DEP for four weeks (total of 18 biological replicates). The experimental design was as follows: dose-response experiment: 0 µg/ml (*n* = 3; control), 3 µg/ml (*n* = 3), 10 µg/ml (*n* = 2), 30 µg/ml (*n* = 2); main experiment: 0 µg/ml (*n* = 4; control), 30 µg/ml (*n* = 4). The genomic DNA of the cells exposed to DEP for 4 weeks was extracted using the DNeasy Blood & Tissue Kit (Qiagen, Hilden, Germany). The concentration of the extracted genomic DNA was measured using a Qubit fluorometer (Thermo Fisher Scientific, Waltham, MA, USA). Epigenome-wide DNA methylation profiling was performed using the Infinium MethylationEPIC BeadChips (Illumina, San Diego, CA, USA), according to the manufacturer’s protocol (for the dose-response experiment) or by outsourcing to Macrogen Japan (Kyoto, Japan) (for the main experiment). Briefly, after performing bisulfite treatment using the EZ DNA Methylation Kit (Zymo Research, Irvine, CA, USA), the processed DNA samples were hybridized, and fluorescently labeled on the Infinium MethylationEPIC BeadChips. The labeled beads were visualized using the iScan system (Illumina, San Diego, CA, USA), and the DNA methylation status of each sample was quantified for each target-specific probe based on the fluorescence intensity.

### Differential methylation analysis

Raw microarray data in idat format were imported into the R programming environment (v.4.1.0) and processed using the *minfi* package (v.1.38.0) [25]. Annotations for each CpG site were added using the *getAnnotation()* function and the *IlluminaHumanMethylationEPICanno.ilm10b4.hg19* package. The quantile method was used to normalize the data. As a quality control (QC), probes with detection *p*-value > 0.01 in one or more samples, probes on sex chromosomes, and probes with a single-nucleotide polymorphism at the CpG site were excluded from subsequent analyses. The β-value and M-value for each CpG site were obtained using the *getBeta()* and *getM()* functions, respectively. Principal component analysis and its visualization were performed using the *plotMDS()* function with the “*gene.selection = common*” parameter. Probe-wise differential methylation analysis for each pair of treated groups was performed using the R package *limma* (v.3.48.0) [26]. The M-value and batch-adjusted design matrices were subjected to the *lmFit()* function to fit the linear model. The resulting model was fit to a given set of contrasts with the *contrasts.fit()* function, followed by the empirical Bayes adjustment *eBayes()* function to obtain *p*-values for the respective CpG sites. *P*-values were adjusted for multiple testing using the Benjamini–Hochberg procedure. An adjusted *p*-value < 0.05 was considered statistically significant. In addition, Bonferroni correction was applied in the dose–response experiment with three pairwise comparisons (control vs. groups exposed to 3, 10, 30 µg/ml DEP). The final threshold for the adjusted *p*-value in the dose–response experiment was set at 0.05/3 = 0.016667.

### RNA isolation and RNA sequencing (RNA-seq)

RNA was isolated from the cells exposed to DEP for four weeks in the main experiment using the RNeasy Mini Kit (Qiagen, Hilden, Germany) following the manufacturer’s protocol (control (*n* = 4); DEP 30 µg/ml (*n* = 4)). The extracted total RNA was quantified and qualified with the NanoDrop (Thermo Fisher Scientific), the Qubit RNA Assay (Thermo Fisher Scientific) and the TapeStation RNA ScreenTape (Agilent Technologies, Inc., Santa Clara, CA, USA). RNA-seq libraries were generated from 500 ng of total RNA for samples with RNA integrity numbers > 8. Briefly, the NEBNext Poly(A) mRNA Magnetic Isolation Module and the NEBNext Ultra II Directional RNA Library Prep Kit for Illumina (New England BioLabs, MA, USA) were used for polyA RNA selection and subsequent RNA-seq library preparation, respectively, according to the manufacturer’s instructions. The RNA-seq libraries from each sample were pooled in equimolar amounts and sequenced on the Illumina NovaSeq 6000 (Illumina), yielding approximately 20 M paired-end reads (2 × 150 bp) per sample. Library preparation and sequencing were conducted by Azenta Life Sciences (Tokyo, Japan).

### Differential expression analysis

The raw fastq files obtained from RNA-seq were trimmed to remove adapters and bases with < 20 quality. STAR aligner [27] was used with the Ensembl_Homo_sapiens.GRCh37 reference genome to align the filtered reads and generate alignment files (bam files). Alignment metrics for the bam files were evaluated using the Samtools software (v.1.3.1) (http://samtools.sourceforge.net), and the results were included into Additional file 4: List 1. HTseq-union was used for gene counting [28]. Genes with counts per million less than 0.5 in more than 4 samples were filtered out from the subsequent differential expression analysis. Afterwards, trimmed mean of M-values (TMM) normalization [29, 30] and log_2_ transformation were applied to obtain a normalized gene count (expression) matrix. Differential analysis was performed using the *limma* workflow with *trend* adjustment. The genes with Benjamini–Hochberg adjusted *p*-value < 0.05 and |log_2_ fold-change (logFC)| > 0.6 were considered to be differentially expressed.

### Verification of RNA-seq results using microarray analysis

To ensure reproducibility of the transcriptional changes in the main experiment, total RNAs for microarray analysis were extracted from five samples of A549 cells exposed to 0 (*n* = 2) or 30 µg/ml (*n* = 3) concentration of DEP for four weeks using the RNeasy Mini Kit (Qiagen, Hilden, Germany), independently of the main experiment. For each sample, total RNA was converted to cyanin-3-labeled cRNA using the Low Input Quick Amp Labeling Kit (Agilent Technologies) according to the manufacturer’s instructions. The labeled cRNA was used for hybridization to the Agilent SurePrint G3 Human GE Microarray 8 × 60K (v.3.0). After hybridization, the microarray slide was washed, and then scanned with an Agilent DNA microarray scanner according to the manufacturer’s protocol. Fluorescence signal intensity was quantified from the scanned image using Feature Extraction software (Agilent Technologies). The resulting signal intensity files were imported into the R programming environment (v.4.1.0) and processed using the *limma* package (v.3.48.0) [26]. Briefly, signal intensities were background corrected (*method = ‘normexp’*), normalized (*method = ‘quantile’*), filtered for failed probes, and used for further differential expression analysis. The differential expression analysis was performed in the same way as the probe-wise differential methylation analysis above, using linear model fitting and empirical Bayes adjustment to obtain *p*-values for the respective genes. The genes with Benjamini–Hochberg adjusted *p*-value < 0.05 and |logFC| > 0.6 were considered to be differentially expressed.

### Enrichment analysis

Region-based enrichment analysis was performed using the *runLOLA()* function of the LOLA R package (v.1.24.0) [31]. This function used one-sided Fisher’s exact test and required three files as input: query regions of interest, universe region set, and region set database. For regulatory region enrichment analysis and TFBS enrichment analysis, the list of genomic positions of the examined CpG sites (all differentially methylated CpGs (DMCs); hypomethylated DMCs (∆β < 0); hypermethylated DMCs (∆β > 0); positively correlated DMCs; negatively correlated DMCs) was provided as query regions of interest. As the universe region set, we used either all QC-passed CpG sites or all DMCs identified in the main experiment. Genomic positions of the regions tested for enrichment were extracted from various databases. Specifically, the genomic positions of CpG islands were downloaded from UCSC ftp using the hg19 genome reference. The genomic positions of DNase hypersensitive sites (DHSs) broad peaks for A549 cells were obtained from the ENCODE database (GEO accession numbers: GSM736580, GSM736506). To call the genomic positions of the peaks associated with H3K9me3, H3K9ac, H3K4me3, H3K4me1, H3K36me3, H3K27me3, H3K27ac marks in A549 cells, raw ChIP-seq fastq files were downloaded from European Nucleotide Archive (study accession number: PRJDB2453; run accession numbers: DRR016932, DRR016933, DRR016934, DRR016935, DRR016936, DRR016937, DRR016938, DRR016939 (input)). Subsequently, the ChIP-seq data were pre-processed, and the genomic positions of the broad or narrow peaks below the *q*-value threshold of 0.01 were called using the MACS3 [32]. The genomic positions of the TFBSs in A549 cells were downloaded from the ENCODE database (wgEncodeRegTfbsClusteredWithCellsV3_hg19.bed). After applying the *runLOLA()* function to the entire set of files, regions of interest with a *q*-value less than 0.05 were considered as DMC enriched.

To compare the transcriptional changes obtained in the main and verification experiments, Gene Set Enrichment Analysis (GSEA) [33] was performed using the GSEA software (v.4.1.0) with default settings except for the following parameters: *“Collapse/Remap to gene symbols”* was set to *“No collapse”* and *“Permutation type”* was set to *“Gene set”.* The input data matrix consisted of normalized log2(TMM) gene expression values obtained from the main RNA-seq experiment. Significantly up- or downregulated genes obtained in the verification experiment were used as predefined gene sets. The degree to which genes in the up- or downregulated gene set were overrepresented at the top or bottom of the logFC-based ranked list of genes from the RNA-seq experiment was assessed using the enrichment score (ES) and its probability at a 5% significant level.

The WebGestalt tool [34] was used to perform gene-based overrepresentation analysis. In brief, the GMT file containing the Hallmark gene sets [35] was downloaded from the Human Molecular Signatures Database (https://www.gsea-msigdb.org/gsea/msigdb/collections.jsp), and then the Senescence gene set [36] was added to the downloaded GMT file. The GMT file containing the ENCODE ChIP-seq transcription factor target gene sets was downloaded from the ChEA3 website (https://maayanlab.cloud/chea3/). Each GMT file, along with lists of differentially expressed genes (DEGs) and reference genes (14,604 genes that passed quality control), was uploaded to the WebGestalt for the overrepresentation analysis. The minimum and maximum number of genes for the gene set were set to 5 and 2000, respectively. Gene sets with Benjamini–Hochberg adjusted *p*-value of less than 0.05 were considered to be significantly enriched.

### Correlation analysis

Correlations between DNA methylation changes in the main and dose-response experiments, as well as transcriptional changes in the main and verification experiments were examined using the *cor.test()* R function with the “*method = spearman*” parameter.

To assess the correlation between DNA methylation and gene expression in the main experiment, *cis-*CpG–gene pairs were generated based on the genomic locations of the CpG site and the transcript start site (TSS) of the gene. The location of the CpG site was determined based on the Illumina Human Methylation EPIC annotation; the TSS location of the gene was determined based on the Ensemble_Homo_sapiens.GRCh37.87.gtf annotation, taking into account the direction of the transcription. A *cis-*CpG–gene pair was formed only if the TSS of the gene was located within a 1-Mbp window to the left or right of the CpG site location. A CpG site paired with a gene in this manner was termed a *cis*-CpG. To calculate the correlation between the DNA methylation level and gene expression of each *cis-*CpG–gene pair, the *cor.test()* R function with “*method = kendall*” parameter was used for a set of DNA methylation (M-values)–expression (normalized gene counts) pairs obtained from eight samples (four controls and four DEP-treated). To find significantly correlated pairs, the significance threshold was set at a nominal *p*-value < 0.001 and the correlation coefficient |r_k_| > 0.9. For significantly correlated *cis-*CpG–gene pairs, *cis-*CpG sites with a correlation coefficient of r_k_ > 0.9 or < -0.9 for all paired genes were considered as positively or negatively correlated, respectively.

### Additional statistical tests

One-way analysis of variance (ANOVA) was performed using the GraphPad Prism version 9.0.1 for Windows (GraphPad Software, Boston, Massachusetts, USA). Linear regression analysis was performed using the *lm()* R function with default settings.

Heatmaps for DNA methylation and gene expression data were generated using the *Heatmap()* R function with default settings.

## Results

### Identification of sufficient DEP dose

To evaluate the effects of DEP on A549 cells at the transcriptomic and DNA methylomic levels, we first determined the DEP concentration that sufficiently induced significant genome-wide DNA methylation changes after 4 weeks of exposure. The dose range of 0, 3, 10 and 30 µg/ml was evaluated.

Through the use of Infinium MethylationEPIC BeadChips, we were able to determine the methylation levels at 850 K CpG sites across the genome. After quality control, normalization, and filtering, 815,486 CpG sites were included in the dose–response analysis. Two methods were used to calculate the methylation level at each CpG site: the *β*-value and the M-value. *β*-values ranging from 0 to 1 were used for data visualization, while M-values were employed for statistical analysis because they have a higher statistical power [37].

**Fig. 1:**
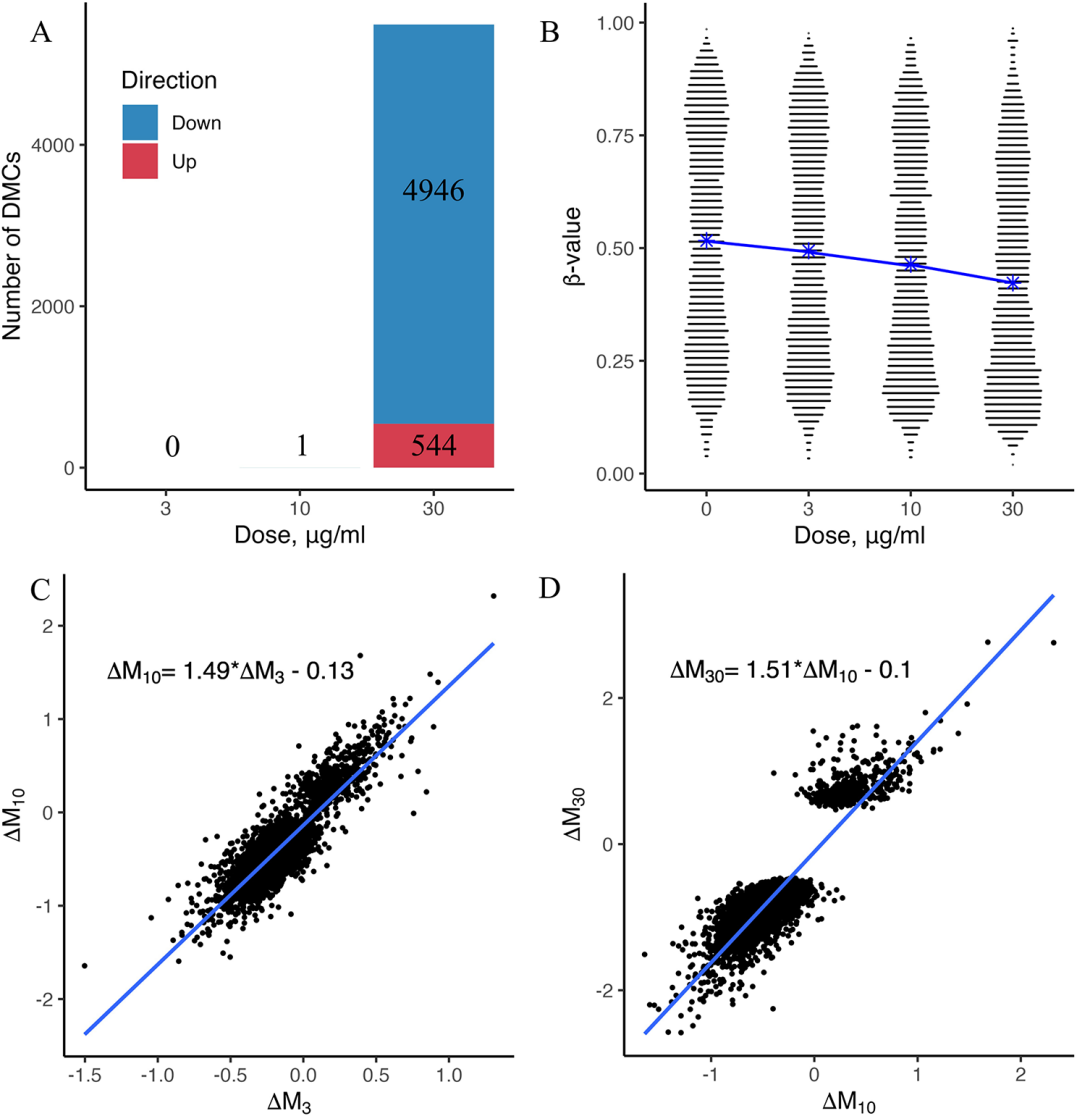
**A** Number of differentially methylated CpG sites (DMCs) after 4 weeks of exposure to diesel exhaust particles (DEP) as a function of DEP concentration. DMCs were obtained from differential methylation analysis using DNA methylation from A549 cells treated with 0 (*n* = 3), 3 µg/ml (*n* = 3), 10 µg/ml (*n* = 2), and 30 µg/ml (*n* = 2) concentrations of DEP. **B** Violin plot of β-values at DMCs as a function of DEP concentration. The blue line connects the mean β-values in each group. **C**–**D** Scatter plots (with linear regression and regression equation) showing the relationship between the DNA methylation levels of the respective groups at each DMC. For each DMC, ∆M_*i*_ = M_*i*_ - M_*control*_, where *i* = 3, 10, 30 µg/ml DEP

The results of principal component analysis revealed that DEP dose explained 18% of the variability in methylation levels as captured by the first principal component (Principal Component 1); hence it was the largest source of variation (Additional file 1: Figure S1). The results of one-way ANOVA applied to the M-value matrix of 815,486 CpG sites showed no significant difference (*p* = 0.9989) in the group means among the control and three treatment groups. Statistically significant genome-wide changes in DNA methylation levels were induced in the A549 cells after 4 weeks of exposure to 30 µg/ml DEP (Fig. 1A; Additional file 2). Compared with the control group, a total of 5,490 DMCs — 544 CpG sites with increased methylation level and 4,946 CpG sites with decreased methylation level — were identified in the 30 µg/ml treatment group.

Although no significant DNA methylation changes were observed in the groups treated with 3 and 10 µg/ml DEP, heatmap analysis based on *β*-values showed the presence of dynamic changes in DNA methylation levels of 5490 DMC with increasing concentrations of DEP (Additional file 1: Figure S2). To assess this dynamics, we constructed linear regressions based on the differences in M-values between the control and DEP-treated groups at each DMC for adjacent DEP concentrations (Fig. 1C–D). The coefficients of the regression equations were greater than 1 at 1.49 and 1.51 (*p* < 0.001), indicating that at each DMC, the ∆M (∆M_30_) between the control and 30 µg/ml treatment group was greater than the ∆M (∆M_3_) between the control and 3 µg/ml treatment group and the ∆M (∆M_10_) between the control and 10 µg/ml treatment group. Therefore, a higher DEP dose resulted in more significant methylation changes at the DEP-affected CpGs. Moreover, as the DEP dose increased, the distribution of *β*-values at the DMCs shifted towards the bottom of the violin plot, indicating a tendency towards hypomethylation after DEP exposure (Fig. 1B).

### Methylation changes in A549 cells after 4 weeks of exposure to 30 µg/ml DEP

**Fig. 2:**
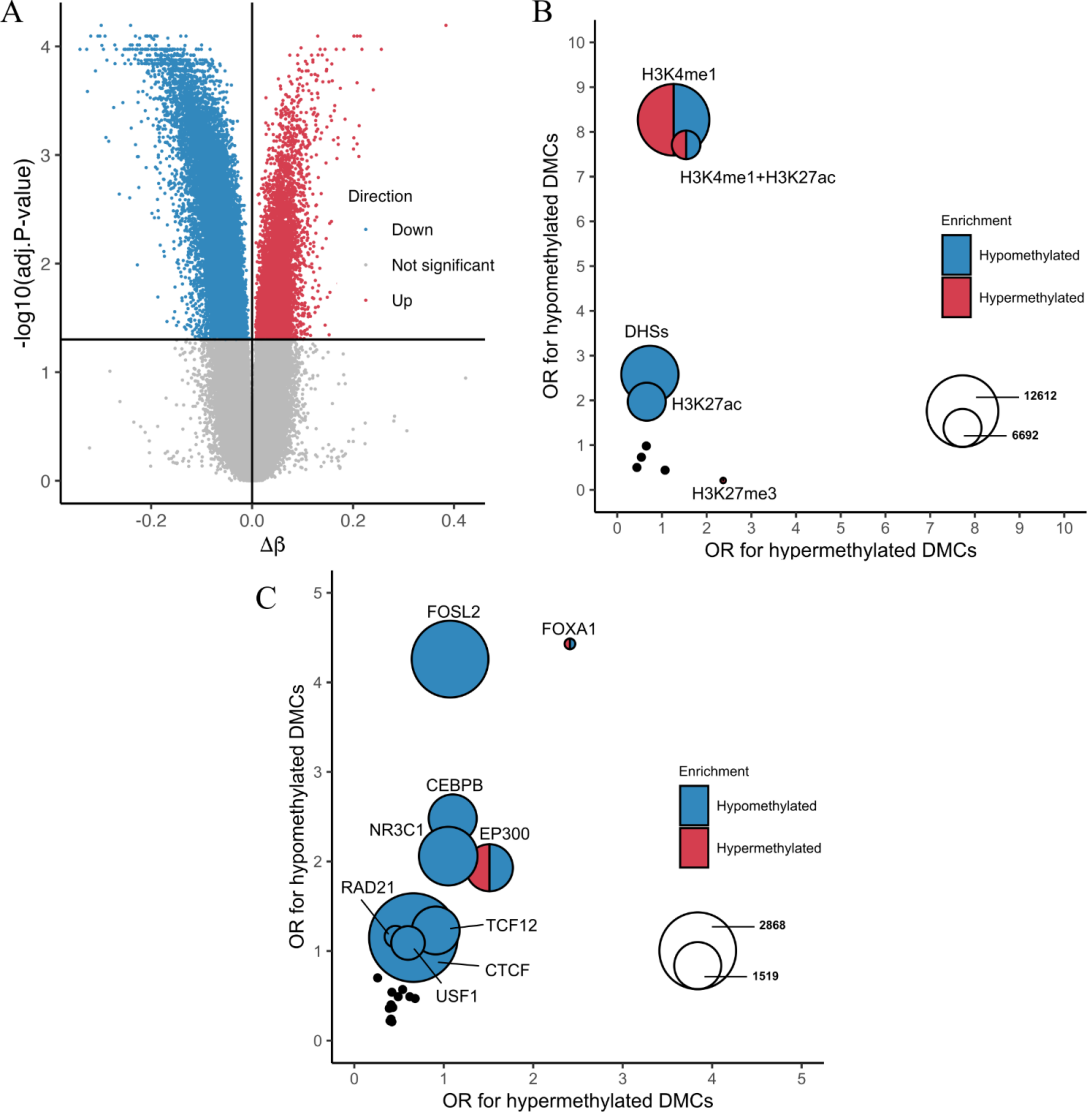
**A** Volcano plot showing the degree of methylation change (β-value difference [Δβ] from control) at each CpG site after 4 weeks of exposure to 30 µg/ml diesel exhaust particles (DEP) relative to the adjusted *p*-value. The horizontal and vertical black lines represent the adjusted *p*-value threshold at 0.05 and ∆β at 0, respectively. The adjusted *p*-values were calculated by differential methylation analysis between the control group (*n* = 4) and the 30 µg/ml DEP-treated group (*n* = 4). **B** Comparison of odds ratios (ORs) representing the degree of enrichment in genomic regions harboring epigenetic marks and CpG islands for each of hypomethylated or hypermethylated differentially methylated CpG sites (DMCs) after 4 weeks exposure to 30 µg/ml DEP. The color of the circle indicates which DMCs (hypomethylated or hypermethylated) are significantly enriched in the respective regions. The size of the circle corresponds to the total number of DMCs co-localized with the given enriched genomic region. **C** Comparison of ORs representing the degree of enrichment in transcription factor (TF) binding regions for each of the hypomethylated or hypermethylated DMCs after four weeks exposure to 30 µg/ml DEP. The color of the circle indicates which DMCs (hypomethylated or hypermethylated) significantly enriched in the respective regions. The size of the circle corresponds to the number of DMCs that overlap with the given enriched binding regions of TFs

Since only 30 µg/ml DEP induced statistically significant DNA methylation changes in the dose–response experiment, we further analyzed its effect on A549 cells after 4 weeks of exposure, independent of the dose–response analysis. In the main experiment, single-site DNA methylation levels were also determined using the Infinium Methylation EPIC BeadChips. A total of 815,398 CpG sites remained after quality control, normalization, and filtering. Through probe-wise differential methylation analysis, we identified 33,900 DMCs distributed across the genome (Additional file 1: Figure S3A; Additional file 3: List 1). Of these, 24,464 (72%) DMCs had decreased methylation levels (hypomethylated) and 9,436 (28%) DMCs had increased methylation levels (hypermethylated), compared with those in the control (Fig. 2A). The results were consistent with those of the dose–response experiment, where the majority of the significant CpG sites (4,974 sites, 90.6%) were also significant in the main experiment (Additional file 1: Figure S4A-B). Furthermore, for the CpG sites identified as differentially methylated in the main experiment, there was a high degree of correlation between the DNA methylation changes observed in these two independent experiments, as demonstrated by the Spearman correlation coefficient of 0.84 (*p*-value < 0.001) (Additional file 1: Figure S4C).

According to the Illumina Human Methylation EPIC annotation, the 33,900 DMCs were annotated at or near 9,979 genes. Approximately 40% of these DMCs were located in the gene body, while 16% were within the 1,500 bp of the TSS (Additional file 1: Figure S3B), indicating that they can overlap with promoters and potentially affect gene expression. Additionally, approximately 35% of these DMCs were associated with CpG islands, shelves, and shores (Additional file 1: Figure S3C), whose DNA methylation levels could be highly correlated with gene expression [38]. Although long-term exposure to DEP altered the methylation levels at a significant number of CpG sites, the maximum change did not exceed 38% (∆β = 0.38), and the average change was 5.7% (Fig. 2A; Additional file 1: Figure S5, Figure S6A).

Next, we examined the association of the identified DMCs with various epigenetic marks and potential regulatory regions reported in untreated A549 cells. DHSs, H3K4me1, H3K4me3, H3K9ac, H3K27me3, H3K9me3, H3K27ac and CpG islands along with their shelf regions in A549 cells were tested for DMC enrichment. In general, DEP-induced DMCs were significantly overrepresented in the DHSs (odds ratio (OR) > 1) and simultaneously underrepresented (OR < 1) in the H3K9me3 regions associated with heterochromatin (Additional file 3: List 2), indicating that the changes in DNA methylation levels occurred more frequently in active regulatory regions [39]. Stratified analysis of hypo- and hypermethylated DMCs showed that the enrichment of DMCs in the DHSs was mainly due to the enrichment of hypomethylated DMCs in the genomic regions (OR_hypo_ = 2.59, OR_hyper_ = 0.73) (Fig. 2B; Additional file 3: List 2). Furthermore, both hyper- and hypomethylated DMCs were overrepresented in the H3K4me1 and H3K4me1 + H3K27ac regions. Notably, among the promoter-associated epigenetic marks, only the inactive promoter mark H3K27me3 showed significant enrichment in hypermethylated DMCs.

We also examined the association between DMCs and TFBSs in A549 cells. Enrichment analysis was performed for binding sites of each of the 24 transcription factors (TFs) that were stored in the ENCODE database, using all DMCs, hypomethylated DMCs, and hypermethylated DMCs, respectively. When the enrichment analysis was performed using all DMCs as query regions of interest, the binding sites of six TFs, such as FOXA1, were significantly associated with DMCs (Additional file 3: List 3). In a stratified analysis, hypomethylated DMCs were overrepresented in the binding sites of FOX1A, FOSL2, CEBPB, EP300, NR3C1, TCF12, RAD21, CTCF, and USF1 transcription factors, whereas the binding sites of FOXA1 and EP300 were also enriched in hypermethylated DMCs. (Fig. 2C; Additional file 3: List 3).

### Transcription changes in A549 cells after 4 weeks of exposure to 30 µg/ml DEP

To investigate how long-term exposure to 30 µg/ml DEP affects gene expression in A549 cells, RNA-seq analysis was performed for the DEP-treated and control groups. After quality control and filtering, 14,604 genes remained for further analysis. Based on the gene counts from the RNA-seq analysis, principal component analysis revealed that 89% of the variation in expression levels was explained by DEP exposure, as captured by the principal component 1 (Additional file 1: Figure S7).

**Fig. 3:**
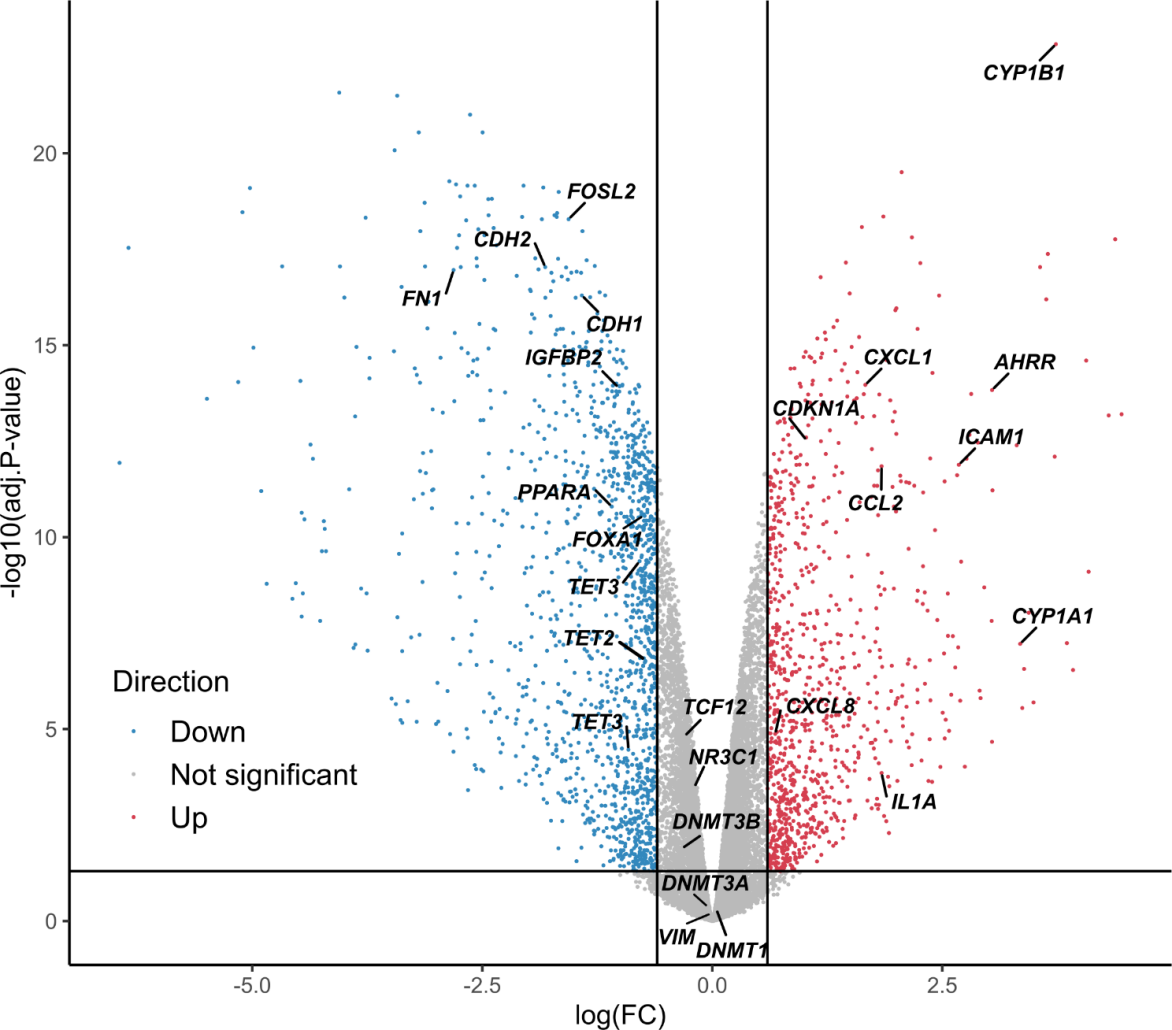
Differentially expressed genes (DEGs) after 4 weeks of exposure to 30 µg/ml diesel exhaust particles (DEP). The volcano plot shows gene expression by log_2_ fold-change (logFC) relative to the adjusted *p*-value. The horizontal and vertical black lines represent the adjusted *p*-value threshold at 0.05 and the |logFC| threshold at 0.6, respectively. The adjusted *p*-values were calculated by differential methylation analysis between the control group (*n* = 4) and the 30 µg/ml DEP-treated group (*n* = 4). Gene symbols indicate the genes mentioned in the paper

Through differential expression analysis, we identified 2,411 DEGs (Fig. 3, Additional file 4: List 2), of which 1,445 were downregulated and 965 were upregulated after 4 weeks of DEP exposure. The expression changes for DEGs showed a high degree of correlation with those observed in the verification experiment (see Additional file 4: List 5), with a Spearman correlation coefficient of 0.774 (*p*-value < 0.001) (Additional file 1: Figure S8C). GSEA also showed significant consistency in the direction of gene expression changes between the two experiments, with enrichment scores of 0.83 and − 0.88 for up- and downregulated genes, respectively (Additional file 1: Figure S8A-B).

**Fig. 4:**
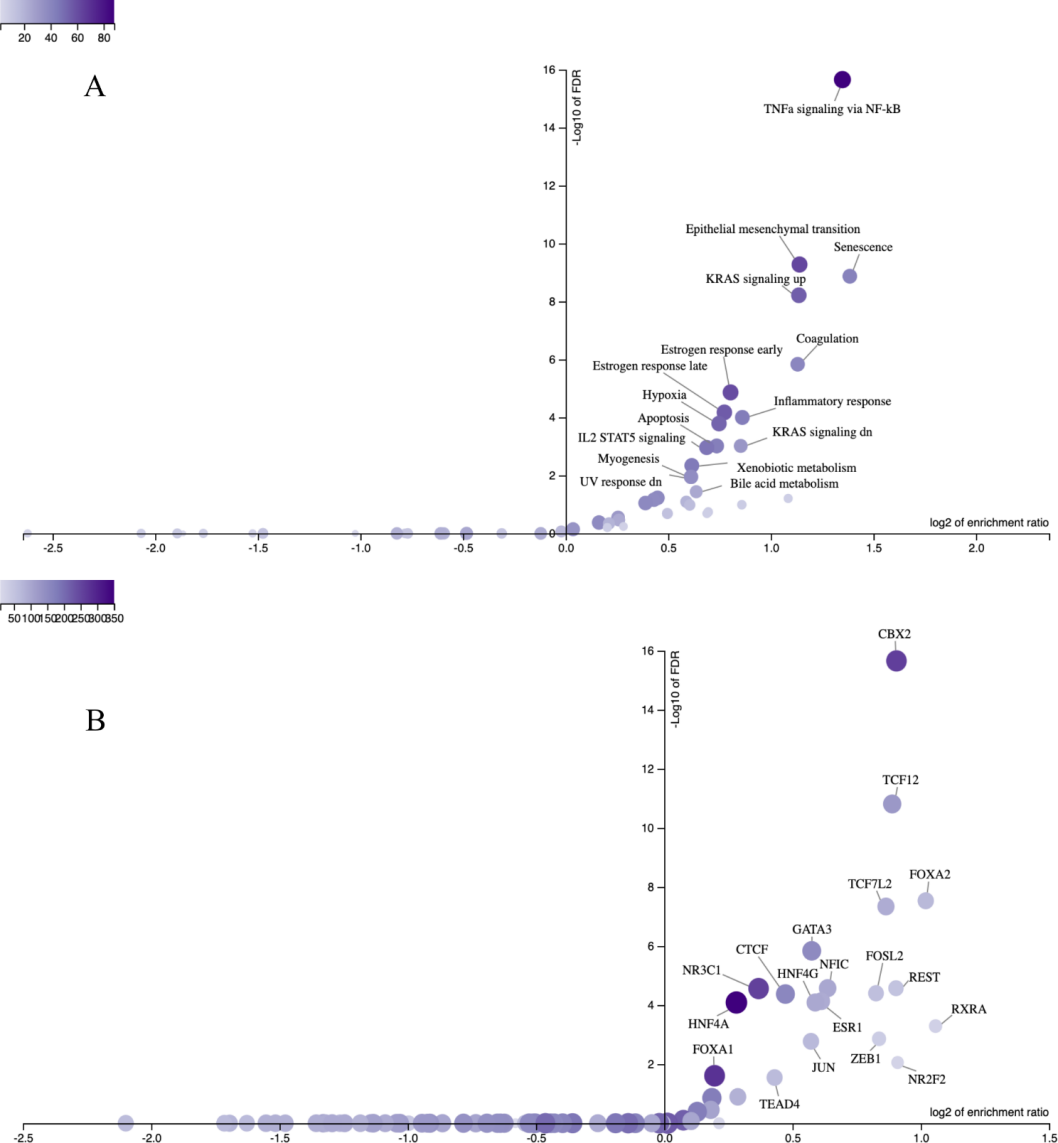
Relationship between enrichment ratio and false discovery rate (FDR) representing the results of overrepresentation of differentially expressed genes (DEGs) using **A** 50 Hallmark gene sets from the Human Molecular Signatures Database with the Senescence gene set and **B** ENCODE transcription factor target gene sets after 4 weeks exposure to 30 µg/ml diesel exhaust particles (DEP). The intensity of the purple color indicates the number of DEGs included in the gene set. The enrichment ratio reflects the number of DEGs included in the gene set of interest divided by the expected number of genes in the gene set, calculated based on the reference genes that passed the quality filter

Overrepresentation analysis applied to the 50 Hallmark gene sets from the Human Molecular Signatures Database [35] helped us to functionally characterize the DEGs. We also tested the association between the DEGs and the SenMayo senescence gene set [36] because senescence plays a key role in initiating airway remodeling, inflammation, and pulmonary fibrosis in the alveolar epithelium [12]. This overrepresentation analysis revealed that DEGs were significantly enriched in 16 gene sets (Fig. 4A; Additional file 4: List 3); among them, *Senescence* had the highest enrichment ratio, followed by *TNF-α signaling via NF-κB*,* Epithelial mesenchymal transition*,* KRAS signaling (up and down)*,* Coagulation*,* Inflammatory response*,* Estrogen response (early and late)*,* Hypoxia*,* Apoptosis*,* IL2 STAT5 signaling*,* Bile acid metabolism*,* Xenobiotic metabolism*,* Myogenesis and UV response (down)* gene sets.

In parallel, we performed an overrepresentation analysis of gene sets containing the target genes of the TFs. Of the 470 TFs from the ENCODE database that were included in the analysis, 19 TFs that could potentially induce the dysregulation of DEGs were identified (Fig. 4B; Additional file 4: List 4), including FOSL2, NR3C1, FOXA1, CTCF and TCF12, whose binding sites were also enriched in hypomethylated DMCs (Fig. 2C; Additional file 3: List 3).

### Concordant DNA methylation and transcriptional changes

The association between changes in DNA methylation and gene expression is of interest because DMCs after DEP exposure in the main experiment were overrepresented in genomic regions associated with gene regulation. Therefore, we further investigated the correlation between DNA methylation levels at DMCs and the expression of nearby genes obtained in the main experiment. We found 106,486 *cis*-DMC–DEG pairs where the TSS of the DEG was located within 1 Mbp on either side of the DMC (*cis*-DMC) (Additional file 5: List 1). Among the 106,486 *cis*-DMC–DEG pairs, a significant correlation between DNA methylation and gene expression was observed for a total of 1,225 pairs (1.15%) formed by 1,179 *cis-*DMCs and 854 DEGs (Additional file 5: List 2). For comparison, we also examined correlated pairs between *cis*-DMCs and genes with no significant expression differences (nonDEGs) within the 1-Mbp window from the DMC and found that the proportion of correlated pairs in the *cis*-DMC–DEG pairs was 2.31 times significantly greater than that in the *cis*-DMC–nonDEG pairs (Additional file 5: List 5). In total, 597 *cis*-DMC–DEG pairs had a negative correlation (correlation coefficient r_k_ < 0), while 628 pairs had a positive correlation (r_k_ > 0) between DNA methylation and gene expression. For the correlated *cis*-DMC–DEG pairs, the distance between the *cis*-DMC and the TSS of the DEG was almost uniformly distributed, taking different values within throughout the entire range of the 1-Mbp window (Additional file 1: Figure S9).

When characterizing the genomic and epigenomic features of the 1,179 DEG-correlated *cis-*DMCs, CpG islands, DHSs, and regions with enhancer-associated epigenetic marks were enriched in DEG-correlated hypomethylated *cis-*DMCs, whereas DEG-correlated hypermethylated *cis-*DMCs were overrepresented in inactive promoters marked by H3K27me3 (Additional file 5: List 3). These enrichment patterns in the stratified analysis were similar to those for all the 33,900 DMCs identified (Additional file 3: List 2). With respect to TFBSs, the binding sites for POLR2A showed a statistically significant overrepresentation in DEG-correlated hypomethylated *cis-*DMCs. In contrast, no TFBS enrichment was observed for DEG-correlated hypermethylated *cis-*DMCs (Additional file 5: List 4). The distribution of positively or negatively DEG-correlated *cis-*DMCs across the different genomic regions was similar, with comparatively equal numbers of positively or negatively DEG-correlated *cis-*DMCs (Additional file 5: Lists 3–4).

## Discussion

In this study, we aimed to investigate the effects of long-term exposure to low-dose DEP on the DNA methylome and transcriptome in human adenocarcinoma alveolar basal epithelial A549 cells, and to evaluate the possible link between methylation levels at individual CpG sites and gene expression. This study has several notable features compared with previous studies on the similar topic. First, we tested lower DEP doses than those previously used for other regions of the respiratory tract because, according to Li et al. [40], the pollutant dose received by the alveolar region is approximately 40 times lower than that received by the tracheobronchial region during inhalation. Second, we integrated two sets of omics data — DNA methylation and gene expression levels, which were quantified through the use of highly accurate and precise Infinium MethylationEPIC BeadChips and RNA-seq. These data allowed us to comprehensively assess the effect of long-term DEP exposure on A549 cells. Furthermore, although the established A549 tumor cell line used can undergo various changes with each cell passage, our results showed good reproducibility, demonstrating a high degree of correlation between the data obtained from independent experiments.

Principal component analysis revealed that DEP exposure accounted for 89% of the variability in gene expression and only 18% of the variability in DNA methylation levels. This finding suggests that the effect of long-term DEP exposure on DNA methylation is less than that on gene expression in A549 cells. Therefore, we only used the adjusted *p*-values without ∆β threshold to identify DMCs because we wanted to detect any minute but stable DNA methylation changes that may directly affect gene expression or may be a by-product of other processes that affect gene expression in response to DEP exposure.

Of the three DEP doses tested in the dose–response experiment, only 30 µg/ml DEP induced a statistically significant changes in DNA methylation levels at many CpG sites after 4 weeks of exposure. Exposure to the lower doses also altered the methylation levels at the DEP-affected CpG sites but to a lesser extent. This finding suggests that different DEP concentrations produce similar effects with different intensities.

After four weeks of exposure to DEP, DNA hypomethylation was observed in A549 cells at most of the DMCs, which is consistent with the finding of Zhang et al. [22] who observed predominant DNA hypomethylation in human bronchial epithelial cells after 24 h of exposure to DEP. In contrast to the findings of Zhang et al. [22], who also reported increased expression levels of *TET1*, *DNMT1*, and *DNMT3A* along with changes in DNA methylation and hydroxymethylation levels after DEP exposure, four-week exposure to DEP in this study resulted in significant downregulation of all the TET family genes (*TET1*, *TET2*, and *TET3*) (Additional file 1: Figure S10A-B) and no marked changes for the DNMT family genes (*DNMT1*, *DNMT3A*, and *DNMT3B*) (Additional file 1: Figure S10C-D). Although the mechanism of action by which DEP exposure causes changes in DNA methylation levels is unknown, the TET and DNMT family genes are known enzymes that may contribute to the alteration of cellular DNA methylation levels. To investigate whether the TET and DNMT family genes are involved in DNA methylation changes after long-term DEP exposure, future studies are needed to determine the detailed time course of changes in DNA methylation and hydroxymethylation levels after DEP exposure using bisulfite and oxidative bisulfite sequencing methods that can distinguish between the types of cytosine modifications (methyl-cytosine and 5-hydroxy-methyl-cytosine). At the same time, the time course of changes in the expression levels and enzymatic activities of the TET and DNMT family genes after long-term DEP exposure needs to be elucidated.

Changes in DNA methylation levels are also known to be associated with the activities of TFs. For example, CTCF can inhibit DNA methylation at unmethylated bound regions or promote hypomethylation at methylated bound regions [41]. The TFBS overrepresentation analysis revealed that there were significant DMC enrichments in several specific TFBSs in A549 cells after four weeks of DEP exposure. Among them, the binding sites of FOXA1 were significantly enriched for both hypomethylated and hypermethylated DMCs at the top ranks. Although it has been reported that FOXA1 induces hypermethylation within the binding regions under the condition of its reduced expression [42], it remains unclear whether the observed enrichments of DMCs within the specific binding regions of TFs including FOXA1 represent changes in DNA methylation levels via the altered TF activities. Future functional analysis of TF activities after DEP exposure will be required to assess the role of TFs in the DEP-induced changes in DNA methylation.

The associations between DNA methylation and transcriptomic levels could provide valuable insights into the mechanism(s) of gene regulation under DEP exposure. Using a multiomics approach, we found 854 DEGs whose expression levels correlated with DNA methylation levels at *cis-*DMCs. Among all *cis-*DMC–gene pairs used, the proportion of *cis-*DMC–DEG pairs was higher than that of *cis-*DMC–nonDEG pairs (OR = 2.31), suggesting that the reported *cis-*DMC–DEG pairs are statistically validated candidates worthy of future experimental verification. In addition, DEG-correlated hypomethylated *cis-*DMCs were overrepresented in DHSs, promoters and enhancers marked by H3K4me1 and H3K27ac, as well as TFBSs of POLR2A, which are highly observed in these regions [43, 44]. This suggests that there may be biological implications behind the correlations of *cis-*DMC–DEG pairs, although the causal relationships between them remain to be elucidated. Further experimental studies are needed to discover biologically coordinated changes in DNA methylation and gene expression after DEP exposure, which may help to identify the environmentally induced regulatory elements [17] involved in the development of lung diseases.

At the transcriptomic level, four weeks of DEP exposure to A549 cells resulted in altered expression of genes associated with target gene sets for 19 TFs as well as 16 biologically predefined gene sets, including *Xenobiotic metabolism*,* Inflammatory response*, *Senescence*, and *Epithelial mesenchymal transition*. The upregulated DEGs included *CYP1A1*,* CYP1B1*, and *AHRR* (Additional file 1: Figure S6B, Figure S10E-F), which are involved in the xenobiotic metabolism and exhibit elevated expression in response to various air pollutants [45–47]. The expression levels of several cytokines, such as *IL1A*, *CCL2*, *CXCL1*, *CXCL8*, and *ICAM1* were also significantly upregulated (Additional file 1: Figure S10G-H), leading to the enrichment of the *Inflammatory response* gene set [48–50]. With respect to the DEG-enrichment of the *Senescence* gene set, we found transcriptional patterns associated with senescence after DEP exposure. Namely, similar to pulmonary fibrosis, the expression of *PPARA* and *IGFBP2* significantly decreased, and that of the senescence marker p21 (*CDKN1A*) increased in the A549 cells exposed to DEP (Additional file 1: Figure S10I-J). These changes could lead to the formation of a senescent state in cells [51]. In the alveolar epithelium, senescence and inflammation are known to be associated with airway remodeling and pulmonary fibrosis [12, 52]. Interestingly, we also have identified significant DEG-enrichments of target gene sets for five TFs, TCF12, ZEB1, NR3C1, TEAD4 and JUN, which were previously reported to be the regulators in idiopathic pulmonary fibrosis [53]. The formation of pulmonary fibrosis in the alveolar epithelium is also associated with epithelial-mesenchymal transition (EMT) [54, 55]. Although the *Epithelial mesenchymal transition* gene set was among the enriched DEGs, the expression levels of key mesenchymal marker genes N-cadherin (*CDH2*) and fibronectin (*FN1*) were significantly decreased in the DEP-treated cells (Additional file 1: Figure S10K-L), which is contrary to the expected outcome (increased expression) during EMT [56]. This result is consistent with our overall findings that significant morphological changes were not detected in the A549 cells exposed to 30 µg/ml DEP for 4 weeks (data not shown). Further studies with longer-term exposure to DEP are required to evaluate whether DEP can induce EMT and senescence, as well as to assess the ability of DEP to influence the activities of TFs. This would help to understand the mechanisms of DEP-induced fibrosis formation.

It is important to note that our analysis has limitations. First, to characterize DNA methylation changes after prolonged exposure to DEP, we used publicly available ChIP-seq data for untreated A549 cells. Due to cell passages, the epigenetic profiles of the cells used may differ somewhat from the available ones. Second, due to the paired nature of DNA methylation and gene expression data and the small sample size, we were forced to use a non-parametric Kendall test with strict significance thresholds to avoid finding false positives for correlated *cis-*DMC–DEG pairs. This resulted in a relatively small number of the correlated *cis-*DMC–DEG pairs identified; thus, a larger sample size is needed to find more true positive associations. Finally, A549 adenocarcinoma alveolar basal epithelial cells may not have the same cellular response to DEP as healthy ATII cells, although the A549 cell line has been widely used as a model for ATII cells. Future studies are needed to investigate whether there are differences in the responsiveness to DEP between the two cell types.

## Conclusions

Long-term exposure of DEP to human adenocarcinoma alveolar basal epithelial cells induced extensive changes at both the transcriptomic and DNA methylomic levels, and these changes may occur through complex and coordinated processes in the cells. Investigating such coordinated changes has a great potential for the discovery of environmentally induced regulatory elements, which allow a more detailed assessment of the mechanisms and magnitude of the effects of any pollutant, including DEP, on the human body.

### Electronic supplementary material

Below is the link to the electronic supplementary material.

Additional file 1

Additional file 2

Additional file 3

Additional file 4

Additional file 5

## Data Availability

DNA methylation (the Illumina Infinium MethylationEPIC BeadChips data in the dose-response and main experiments) and gene expression (RNA-seq data in the main experiment and the Agilent SurePrint G3 Human GE Microarray 8 × 60K v.3.0 data in the verification experiment) datasets supporting the conclusions of this article are available in the Gene Expression Omnibus (GEO) repository with the SuperSeries number GSE267217. Other data that support the findings of the study are available from the corresponding author (AT) upon request.

## Abbreviations

ANOVA: Analysis of Variance
ATI: Alveolar epithelial type I
ATII: Alveolar epithelial type II
DEG: Differentially expressed gene
DEP: Diesel exhaust particles
DHS: DNase hypersensitive site
DMC: Differentially methylated CpG site
EMT: Epithelial-mesenchymal transition
ES: Enrichment score
FBS: Fetal bovine serum
logFC: log_2_ fold-change
GSEA: Gene Set Enrichment Analysis
nonDEG: Gene with no significant expression difference
OR: Odds ratio
PAH: Polycyclic aromatic hydrocarbon
QC: Quality control
RNA-seq: RNA sequencing
TF: Transcription factor
TFBS: Transcription factor binding site
TMM: Trimmed mean of M-values
TSS: Transcription start site
