# Supplementary material for: Long-term exposure to diesel exhaust particles induces concordant changes in DNA methylation and transcriptome in human adenocarcinoma alveolar basal epithelial cells
Source: Epigenetics Chromatin. 2024 Aug 5;17:24. doi: 10.1186/s13072-024-00549-3 (PMC11299313; doi:10.1186/s13072-024-00549-3)
Supplement: Supplementary file 1 — Additional file 1 [file 13072_2024_549_MOESM1_ESM.pdf]

Long-term exposure to diesel exhaust particles induces concordant changes in DNA methylation and transcriptome in human adenocarcinoma alveolar basal epithelial cells

Content

Figures **2**
Figure S1. 2
Figure S2. 3
Figure S3. 4
Figure S4 5
Figure S5. 6
Figure S6. 7
Figure S7. 8
Figure S8 9
Figure S9 10
Figure S10. 11

Tables **12**
Table S1 12

Figures

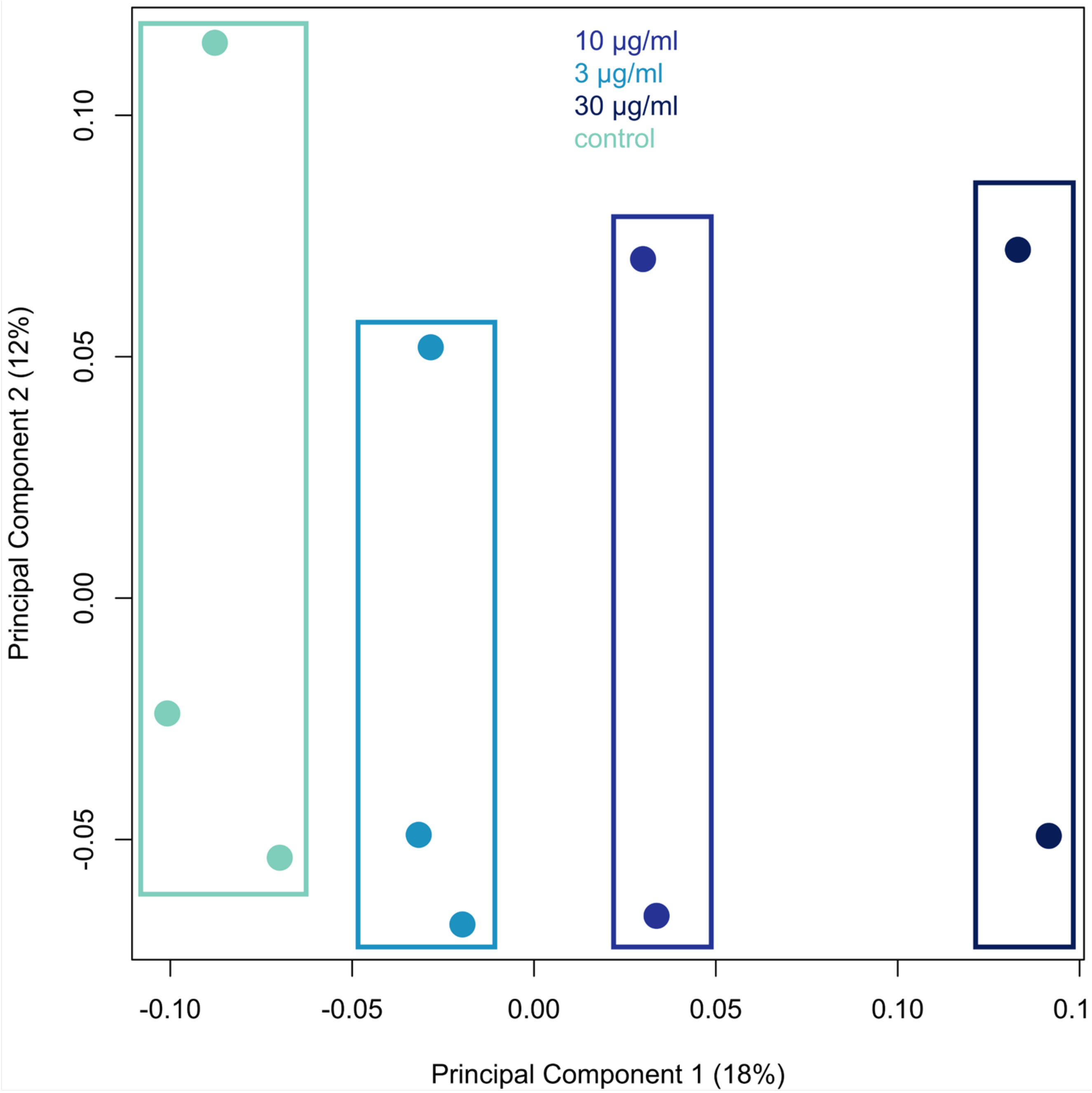

Figure S1. Principal component analysis based on the M-value profiles of 10 samples exposed to diesel exhaust particles at 0, 3, 10, 30 µg/ml concentrations for four weeks.

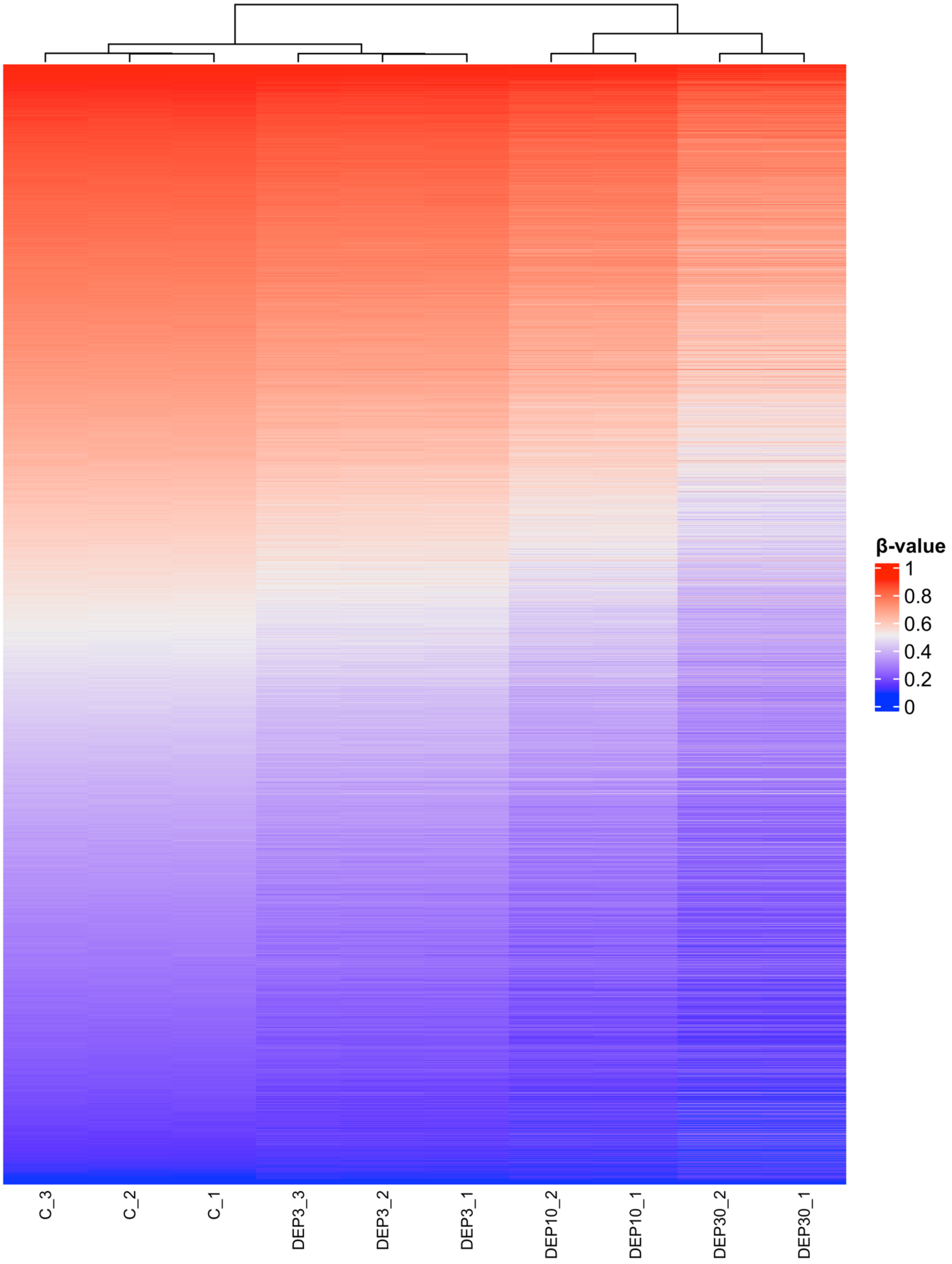

Figure S2. Heatmap of β-values at 5490 differentially methylated CpG sites (DMCs) in the dose-response experiment. The β-values at the DMCs (rows) were measured in A549 cells exposed to diesel exhaust particles (DEP) at concentrations of 0 (shown in columns as C), 3 (DEP3), 10 (DEP10) and 30 (DEP30) µg/ml for four weeks. The β-values were color-coded according to the color scale on the right side of the heatmap.

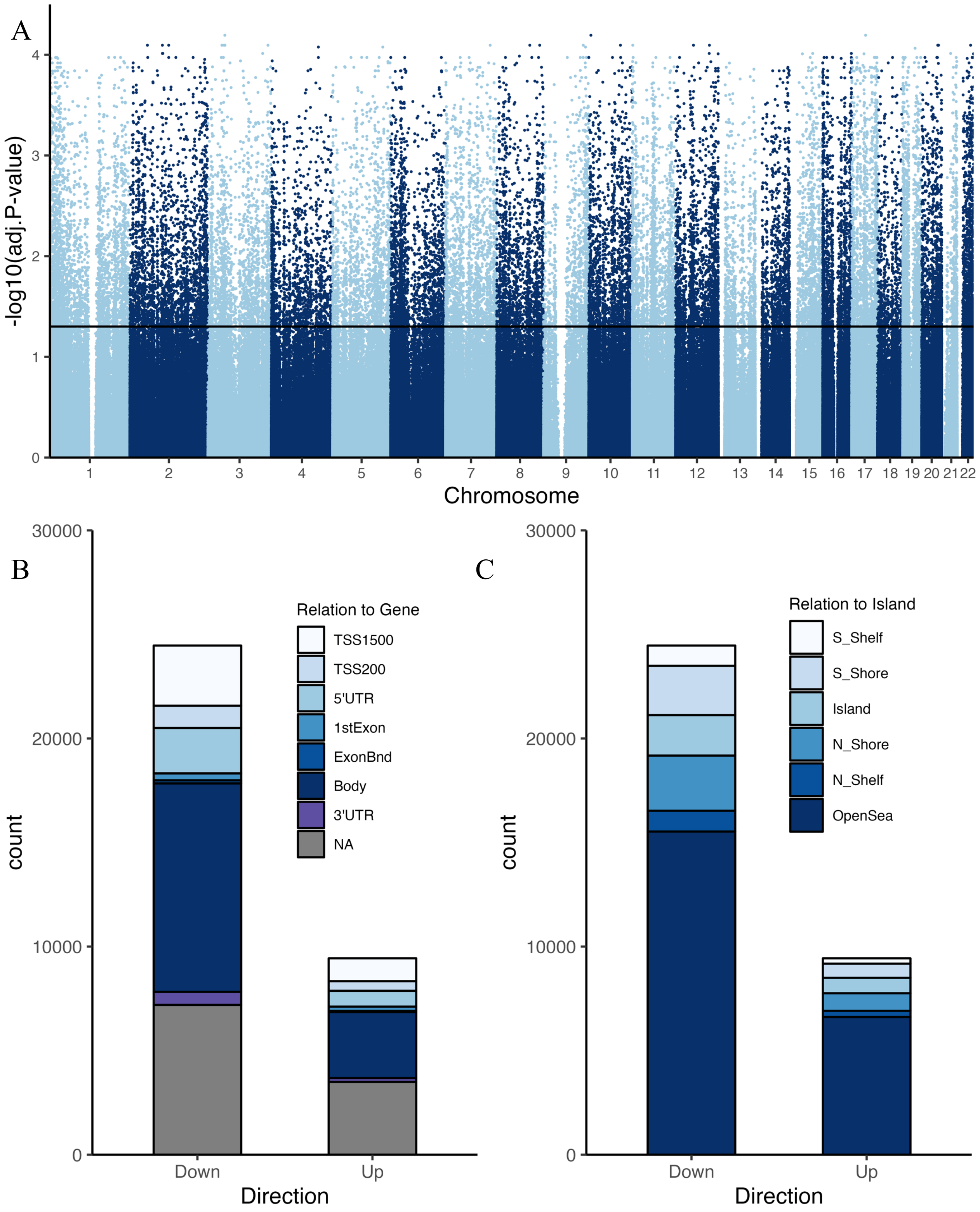

Figure S3. A Manhattan plot of the results of differential methylation analysis comparing DNA methylation levels of A549 cells exposed to diesel exhaust particles for four weeks (n=4) with their controls (n=4) in the main experiment. The horizontal black line intersects the adjusted *p*-value threshold of 0.05. **B** Number of differentially methylated CpG sites (DMCs) broken down by relation to the nearest gene and relative methylation change. TSS1500: 200-1500 bp upstream of the transcription start site (TSS); TSS200: 0-200 bp upstream of the TSS; 5'UTR: 5' untranslated region between the TSS and the ATG start site of the gene; 1stExon: first exon of the gene; Body: region between the ATG start site and the stop codon; ExonBnd: region within 20 bp of the start or end of an exon; 3'UTR: 3' untranslated region between the stop codon and the poly-A tail; NA: not assigned to any gene. **C** Number of DMCs by the closest CpG island and relative methylation change. Island: region > 500 bp length and with > 55% CpGs; N_/S_Shore: region ~2Kb left/right from the island; N_/S_Shelf : ~4Kb left/right from the island.

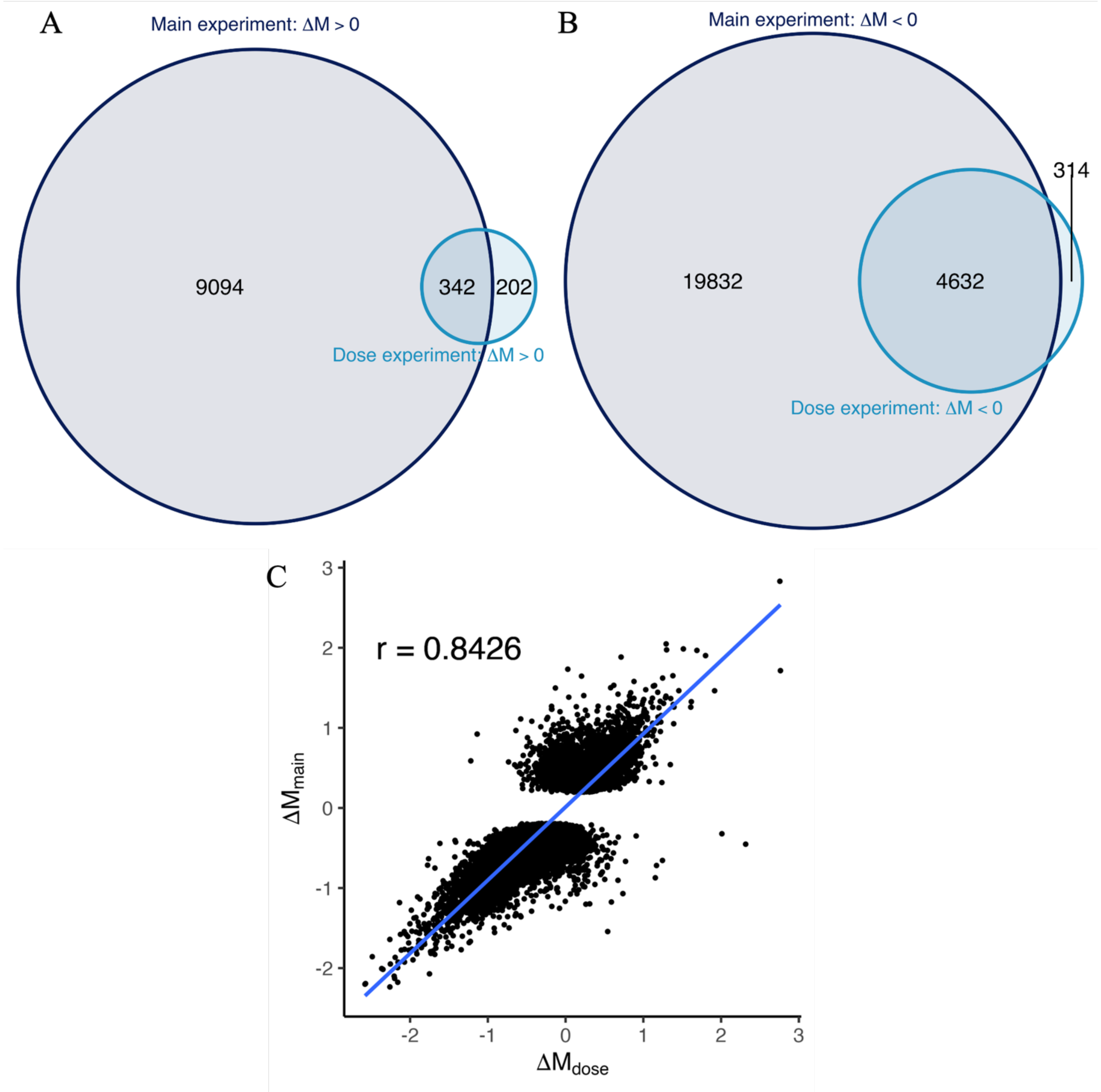

Figure S4. Degree of overlap of differentially methylated CpG sites (DMCs) with (**A**) increased and (**B**) decreased methylation levels in the dose-response and the main experiments, respectively, after four weeks of exposure to 30 μg/ml diesel exhaust particles (DEP) compared to the control. The DMCs identified in the dose-response (n=3 for control and n=2 for DEP-treated samples) and main (n=4 for control and n=4 for DEP-treated samples) experiments are indicated by light blue and light gray circles, respectively. **C** Scatter plot of the mean differences in M-values in the two-group comparison (DEP 30 μg/ml vs. control) of the main experiment (ΔM_{main}) versus those in the dose-response experiment (ΔM_{dose}) at 33,900 DMCs identified in the main experiment. The r value represents the Spearman correlation coefficient, which expresses the degree of correlation between ΔM_{main} and ΔM_{dose}.

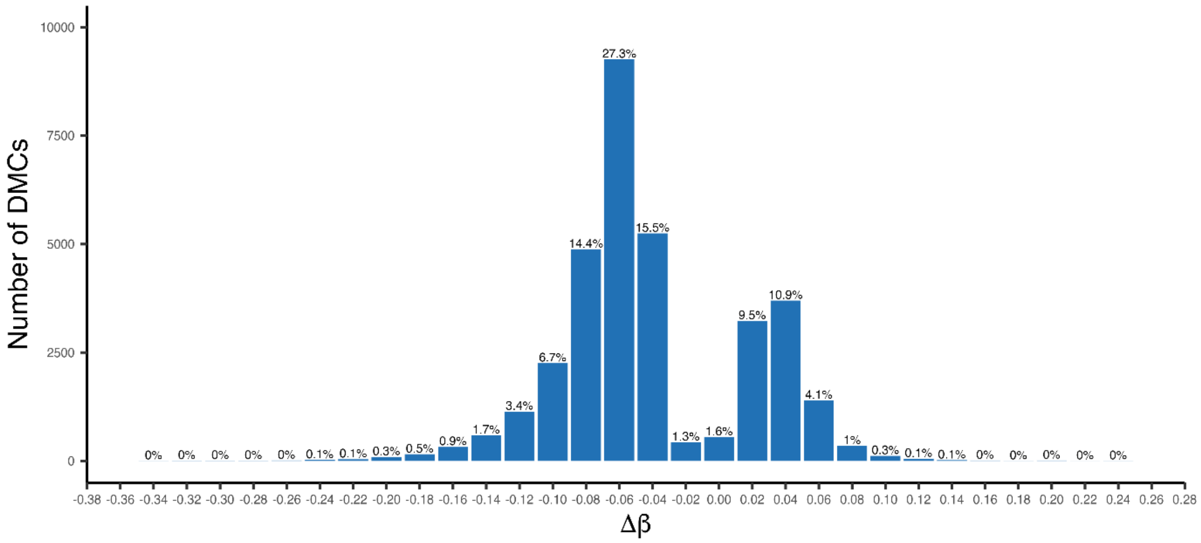

Figure S5. Histogram of Δβ values after four weeks of exposure to 30 μg/ml diesel exhaust particles (n=4) compared to control (n=4) in the main experiment. For each differentially methylated CpG site (DMC), $\Delta\beta = \beta_{30} - \beta_{control}$.

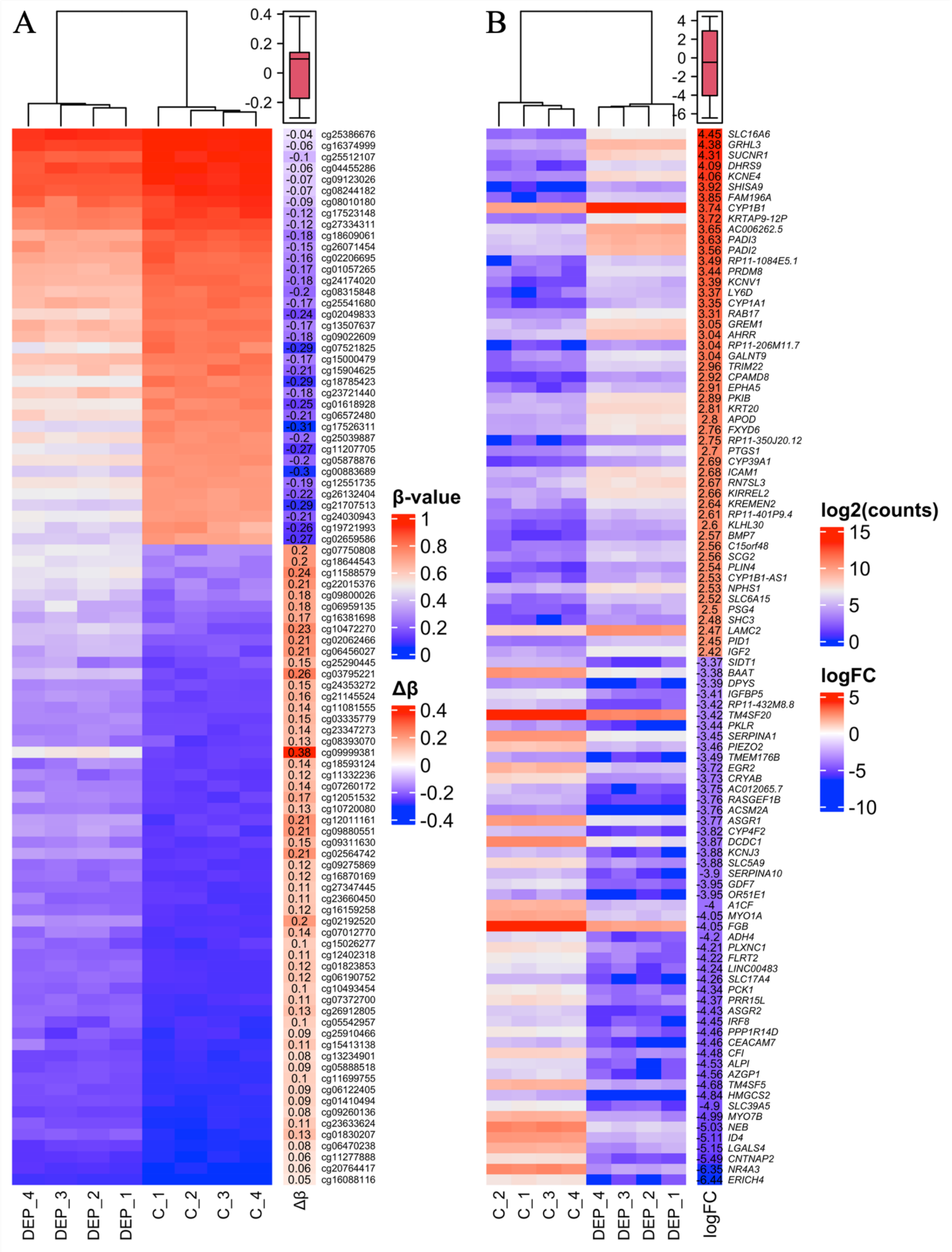

Figure S6. **A** Heatmap of hyper/hypomethylated differentially methylated CpG sites (DMCs) with |logFC|>1.2. Hyper/hypomethylated CpG sites are defined as CpG sites with mean $\beta > 0.7$ or < 0.3 in control samples, respectively. Right panel represents $\Delta\beta$ between case and control for each DMC after 4 weeks of exposure to 30 μg/ml diesel exhaust particles (DEP). **B** Heatmap of the top 100 differentially expressed genes identified after four weeks of exposure to 30 μg/ml DEP compared with the control and sorted by logFC. Log2(counts) represents log2-transformed TMM-normalized gene counts from RNA sequencing analysis.

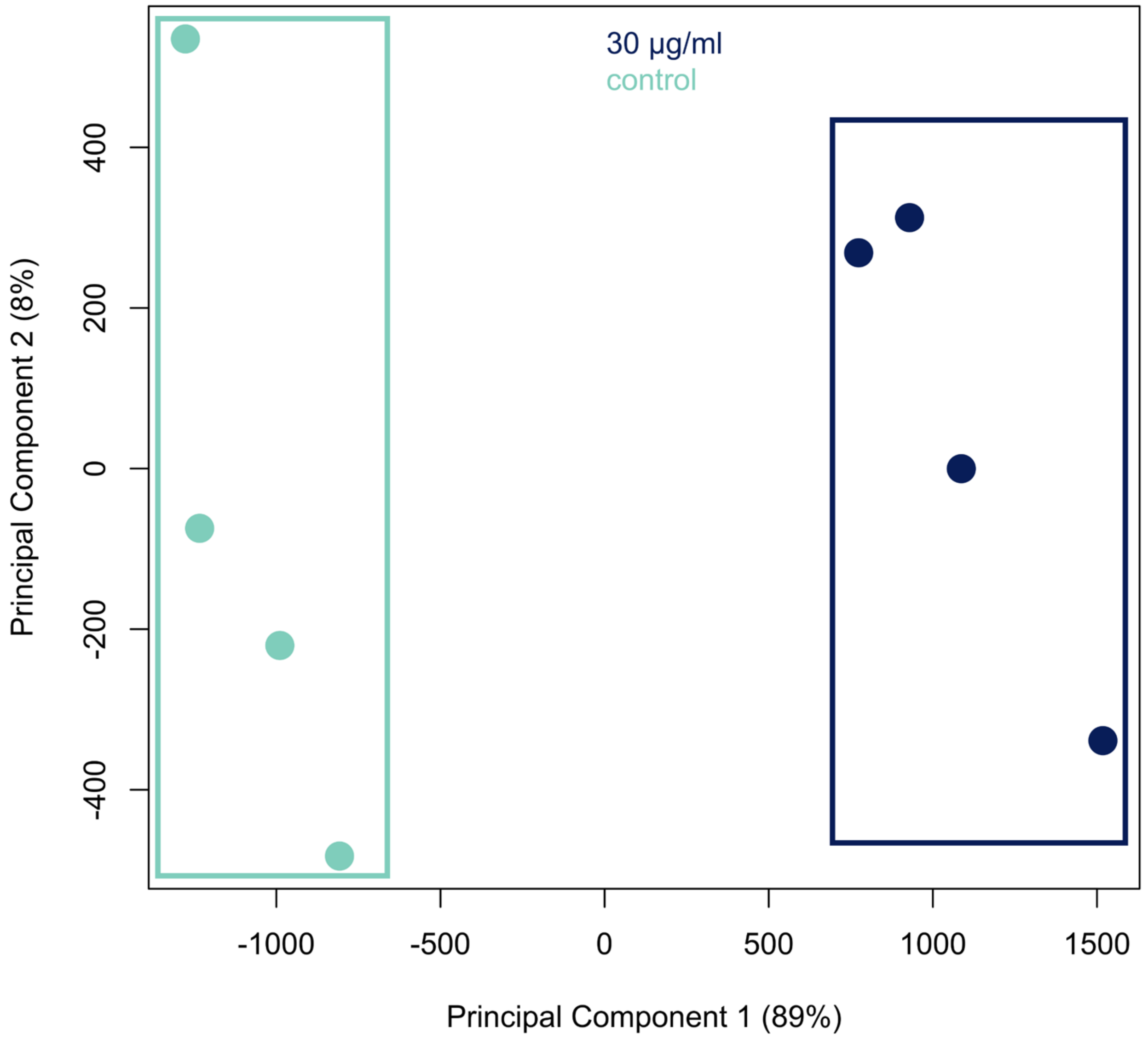

Figure S7. Principal component analysis based on gene count profiles from RNA sequencing of 8 samples exposed to 0 or 30 µg/ml diesel exhaust particles for four weeks.

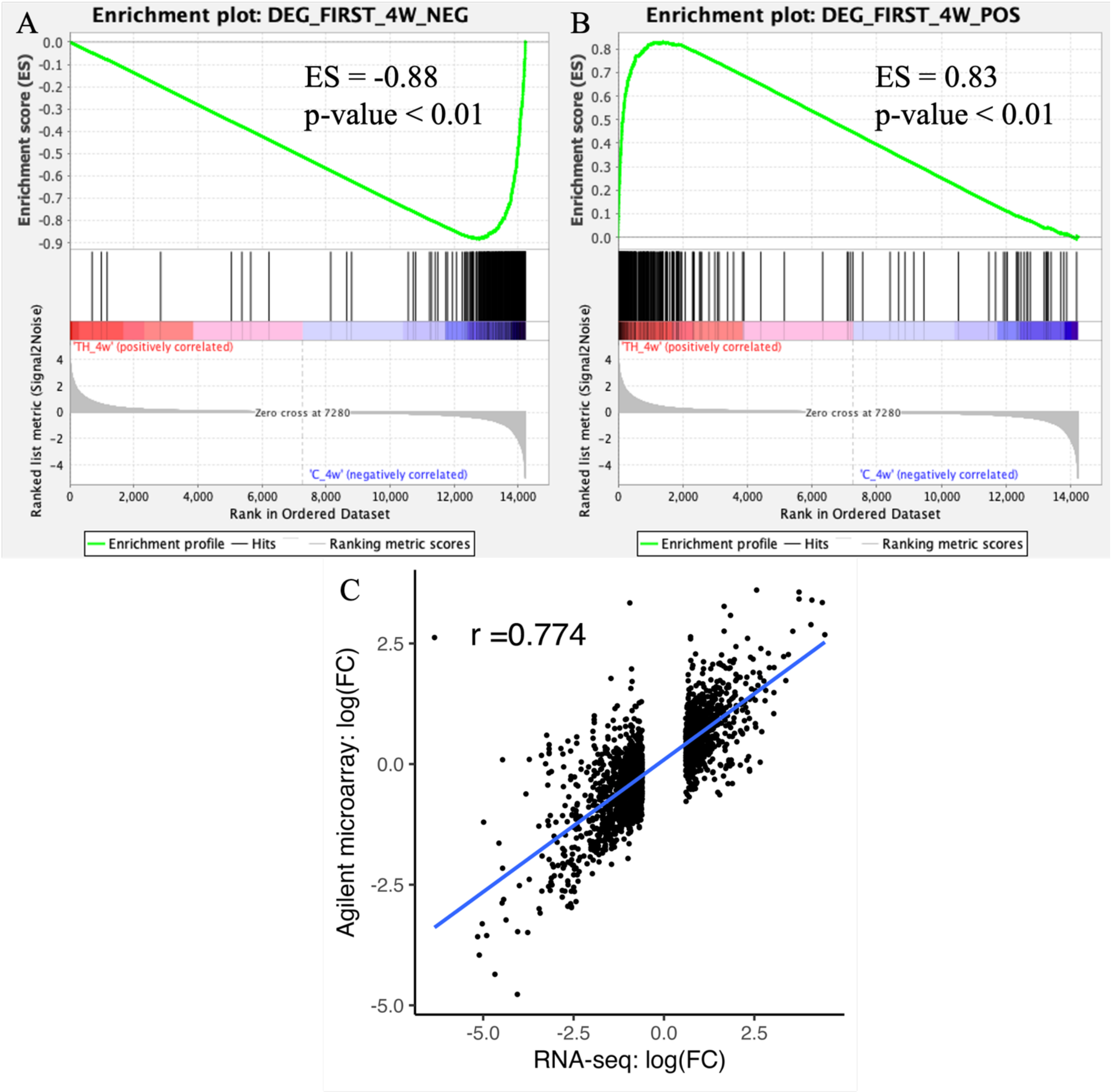

Figure S8. Validation of transcriptional changes in A549 cells exposed to 30 µg/ml diesel exhaust particles (DEP) for four weeks by comparing the results of the two independent experiments (main: n=4 for control and n=4 for DEP-treated groups; verification: n=2 for control and n=3 for DEP-treated groups). Gene Set Enrichment Analysis (GSEA) for RNA sequencing (RNA-seq) data in the main experiment using predefined gene sets consisting of significantly (**A**) downregulated and (**B**) upregulated genes in the validation experiment with the Agilent microarray. Enrichment Score (ES) reflects the degree to which a gene set is overrepresented at the top or bottom of a ranked list of genes. **C** Scatter plot of the log fold-change (FC) values (x-axis) in the two-group comparison (DEP 30 µg/ml vs. control) of the main RNA-seq experiment versus those (y-axis) in the Agilent microarray experiment, for 2,411 differentially expressed genes (DEGs) in the main experiment. The *r* value represents the Spearman correlation coefficient, expressing the degree of correlation between the two logFC values.

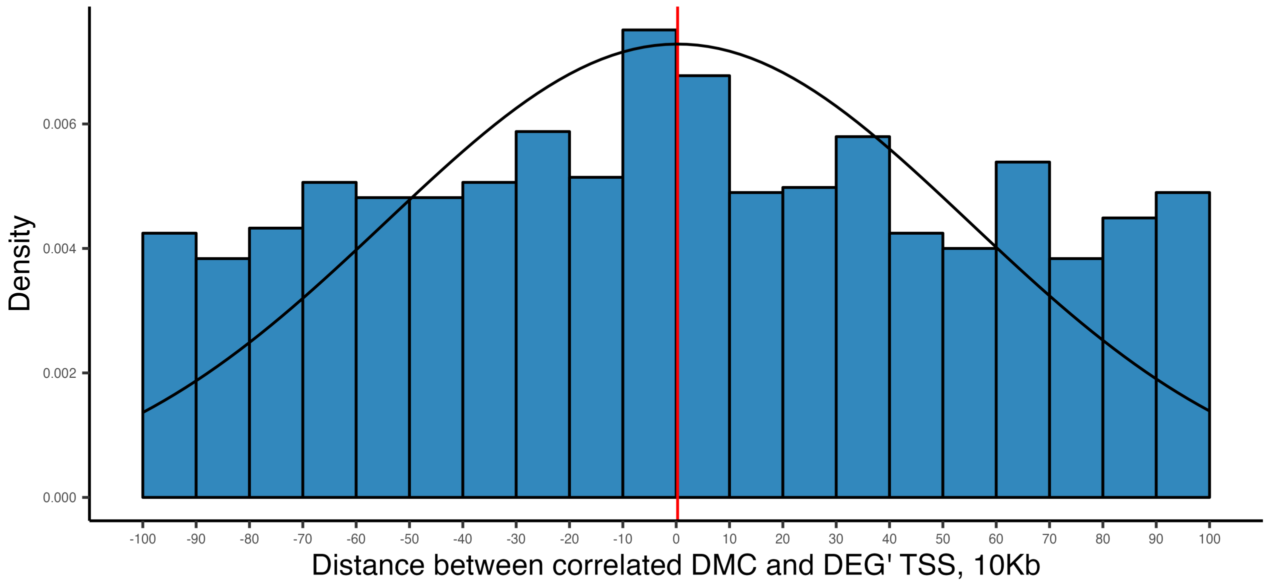

Figure S9. Distribution of physical distances between the differentially methylated CpG sites (DMCs) and the transcription start site (TSS) of differentially expressed genes (DEGs) for each of 1,225 correlated *cis*-DMC–DEG pairs. The red vertical line indicates the median distance.

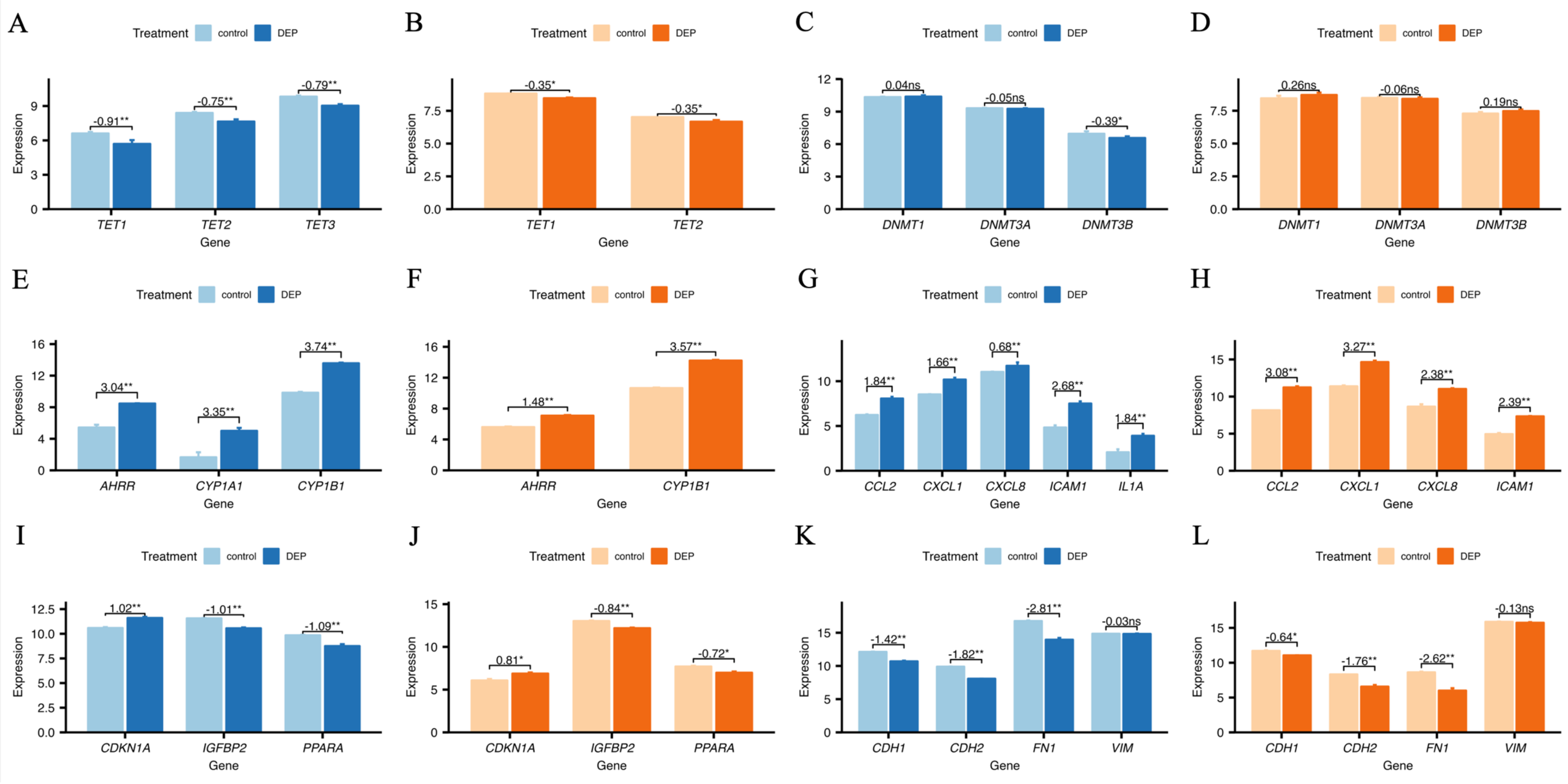

Figure S10. Expression changes of (**A, B**) ten-eleven translocation (TET) family genes, (**C, D**) DNA methyltransferase (DNMT) family genes, (**E, F**) pollutant-related genes, (**G, H**) inflammation-related genes, (**I, J**) early senescence-associated genes and (**K, L**) major epithelial-mesenchymal transition marker genes after four weeks of exposure to 30 µg/ml diesel exhaust particles (DEP) compared to the control. RNA sequencing results from the main experiment (n=4 for control and n=4 for DEP-treated groups) are shown as blue colored bars (panels A, C, E, G, I, K), and microarray results from the verification experiment (n=2 for control and n=3 for DEP-treated groups) are shown as orange colored bars (panels B, D, F, H, J, L). The Y-axis shows the log_2(TMM) value in the main experiment, and the normalized signal intensity value in the verification experiment, respectively. Numbers at top of bars represent logFC; * adjusted *p*-value < 0.05; ** adjusted *p*-value < 0.001; ns - not significant.

Tables

Table S1
Concentrations of polycyclic aromatic hydrocarbons (PAHs) in the diesel exhaust particles used

PAH	$\mu g/g$
Naphthalene	n.d
Acenaphthene	n.d
Fluorene	0.48
Phenanthrene	23.15
Anthracene	1.59
Fluoranthene	24.79
Pyrene	35.40
Benzo(a)anthracene	2.14
Chrysene	n.d
Benzo(b)fluoranthene	2.44
Benzo(k)fluoranthene	0.66
Benzo(a)pyrene	0.80
Dibenzo(ah)anthracene	n.d
Benzo(ghi)perylene	1.94
Indeno(1,2,3-cd)pyrene	1.18

n.d = not detected
